# Complexité de prise en charge d’une macrodactylie au niveau de la main: entre préjudice esthétique et fonctionnel

**DOI:** 10.11604/pamj.2018.30.45.15118

**Published:** 2018-05-18

**Authors:** Mouna Ejjiyar, Saloua Ettalbi

**Affiliations:** 1 Service de Chirurgie Plastique, CHU Mohammed VI, Marrakech, Maroc

**Keywords:** Macrodactylie, main, préjudice fonctionnel, amputation, Macrodactyly, hand, functional injury, amputation

## Abstract

Macrodactylia is a rare congenital malformation of unknown origin that can affect the fingers or the toes. It is characterized by an increase in the size of all the elements of one or more rays, recognizable at birth or occurring in a progressive manner. Social, aesthetic as well as functional impact imposes early surgical management or even amputations in advanced forms. We report the case of a 18-year old unmarried, left handed patient without a profession, admitted to the Department of Burns, Plastic and Aesthetic Surgery at the University Hospital Mohammed VI in Marrakech, with progressive acrodactylia involving the first and the second ray of the right hand. Clinical examination showed hypertrophy of the first two rays of the right hand associated with predominant fibro-fatty infiltration of the palmar as well as phalanx deviation 90 degrees at the level of the second finger. Given the severity of the macrodactylia, amputation of the second ray associated with reduction of the fatty infiltration of the palmar was proposed to the patient, allowing him to maintain pollici-digital pinch and to return to a social life as close as possible to normal life.

## Image en médecine

On définit la macrodactylie comme une malformation congénitale d'étiologie inconnue et d'incidence rare pouvant intéresser les doigts ou les orteils, et caractérisée par l'augmentation, dès la naissance ou de façon progressive, de la taille de tous les éléments d'un ou plusieurs rayons. Son retentissement social, esthétique, et surtout fonctionnel imposent une prise en charge précoce passant par plusieurs interventions chirurgicales voire même des amputations dans les formes évoluées. Nous rapportons le cas d'un jeune patient âgé de 18 ans, célibataire, sans profession, gaucher, admis au service de chirurgie plastique, réparatrice, esthétique et des brûlés du CHU Mohammed VI de Marrakech, pour prise en charge d'une forme progressive de macrodactylie intéressant les premier et deuxième rayons de la main droite. A l'examen, on retrouvait une hypertrophie des deux premiers rayons de la main droite, associée à une infiltration fibro-graisseuse prédominant en palmaire, ainsi qu'une déviation phalangienne en angulation à 90° au niveau du deuxième doigt. Devant cette forme sévère de macrodactylie, une amputation du 2^ème^ rayon associée à une réduction de l'infiltration graisseuse au niveau palmaire a été proposée au patient, lui permettant de conserver une pince pollici-digitale, et de retrouver une vie sociale le plus proche possible de la normale.

**Figure 1:**
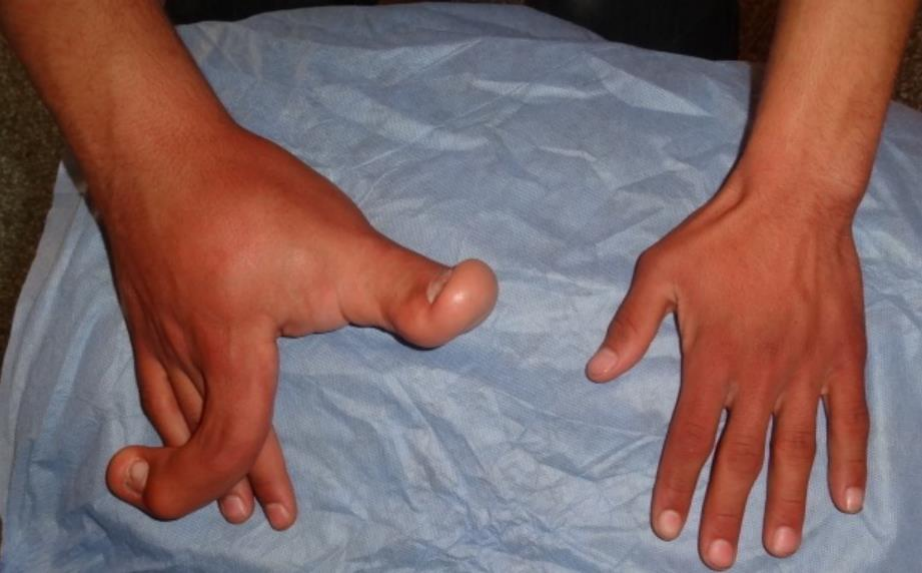
Macrodactylie évoluée de la main droite

